# “Placebo by Proxy” and “Nocebo by Proxy” in Children: A Review of Parents' Role in Treatment Outcomes

**DOI:** 10.3389/fpsyt.2020.00169

**Published:** 2020-03-11

**Authors:** Efrat Czerniak, Tim F. Oberlander, Katja Weimer, Joe Kossowsky, Paul Enck

**Affiliations:** ^1^Department of Pediatrics, BC Children's Hospital Research Institute, University of British Columbia, Vancouver, BC, Canada; ^2^Department of Psychosomatic Medicine and Psychotherapy, Ulm University Medical Center, Ulm, Germany; ^3^Department of Anesthesiology, Critical Care and Pain Medicine, Harvard Medical School, Boston Children's Hospital, Boston, MA, United States; ^4^Department of Clinical Psychology & Psychotherapy, University of Basel, Basel, Switzerland; ^5^Department of Psychosomatic Medicine and Psychotherapy, University Medical Hospital Tübingen, Tübingen, Germany

**Keywords:** placebo effect, nocebo effect, nonspecific effects, treatment environment, clinical implications

## Abstract

The “placebo (effect) by proxy” (PbP) concept, introduced by Grelotti and Kaptchuk (1), describes a positive effect of a patient's treatment on persons in their surrounding such as family members or healthcare providers, who feel better because the patient is being treated. The PbP effect is a complex dynamic phenomenon which attempts to explain a change in treatment outcome arising from an interaction between a patient and an effect from proxies such as parents, caregivers, physicians or even the media. By extension the effect of the proxy can also have a negative or adverse effect whereby a proxy feels worse when a patient is treated, giving rise to the possibility of a “nocebo (effect) by proxy” (NbP), and by extension can influence a patient's treatment response. While this has yet to be systematically investigated, such an effect could occur when a proxy observes that a treatment is ineffective or is perceived as causing adverse effects leading the patient to experience side effects. In this narrative review, we take these definitions one step further to include the impact of PbP/NbP as they transform to affect the treatment outcome for the patient or child being treated, not just the people surrounding the individual being treated. Following a systematic search of literature on the subject using the Journal of Interdisciplinary Placebo Studies (JIPS) database (https://jips.online) and PubMed (NCBI) resulted in very few relevant studies, especially in children. The effect of PbP *per se* has been studied in parents and their children for temper tantrums, acupuncture for postoperative symptoms, as well as for neuroprotection in very preterm-born infants. This paper will review the PbP/NbP concepts, show evidence for its presence in children's treatment outcome and introduce clinical implications. We will also offer suggestions for future research to further our understanding of the role of the proxy in promoting or distracting from treatment benefit in children. Increasing an appreciation of the PbP and NbP phenomena and the role of the proxy in children's treatment should improve research study design and ultimately harness them to improve clinical child healthcare.

## Introduction

To date attention has focused on research studying the placebo effect (PE), the biopsychosocial process, which engages perceptual and cognitive processes that lead to therapeutic benefits associated with the administration of a placebo in the context of individuals being treated (2). These include the impact of factors such as learning, conditioning and the clinical encounter, which affect outcomes, typically via the individual (3–6). In contrast, the placebo effect needs to be distinguished from the placebo response, which includes all health outcomes that follow administration of an inactive treatment (7). The placebo response is widely considered a phenomenon underlying a positive treatment response to both the administration of active medication and treatment with an inert substance (placebo) in a randomized controlled trial, and is related among others to spontaneous remission, regression the mean and the Hawthorne effect (i.e., the effect of being observed) (2).

**Figure 1:**
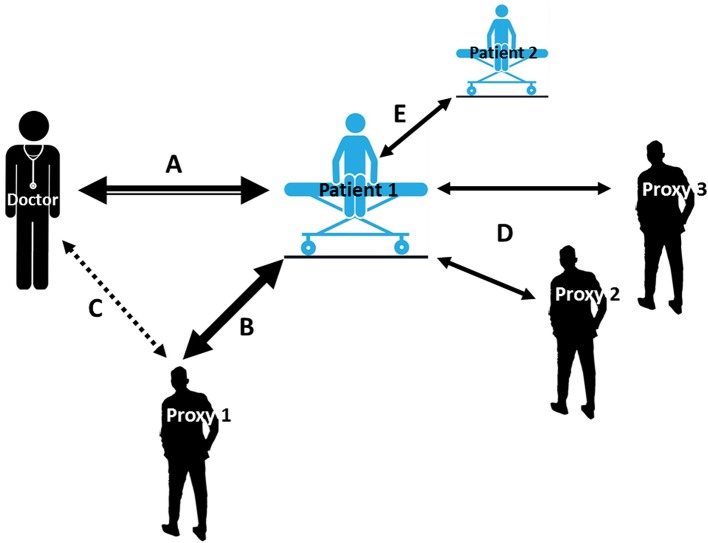
Accumulating and iterative interactions between patient and proxies. **A:** An ideal-world clinical setting where the patient exchanges information only with the physician. The intensity of interaction narrows as more communication channels (e.g., B, C…) open; **A-B:** patient has one proxy in addition to the physician; **A-C:** physician receives reports from both patient and proxy; **A-D:** social environment of patient contains more than one proxy, also proxies with different proximity to the patient including parents, children, siblings, relatives and friends, peers, colleagues; **A-E:** patient has physical/online contact with at least one more patient who has/had a relevant condition, whereby observed treatments and their efficacy become contributors to the overall treatment response.

Many factors may have a direct effect on one's treatment: subjective e.g., expectation of clinical benefit (8, 9), conditioning (10, 11), mood (12, 13), patient-clinician interaction (14, 15) (see Figure 1, Arrow A), age (16), and objective e.g., medication labeling (17) or study design (18–20). The PE may also be the result of patients' mindset guiding their perceptions and thus their interpretation of the clinical environment, affecting behavior e.g., decision-making (21, 22) or driving biological changes e.g., in the immune system (23). Rather than dismissing factors surrounding one's treatment as “nonspecific,” these elements underlying the PE may actually be central to understanding treatment outcomes in general and in children in particular (16, 24).

### Setting the Scene

The placebo response in children has been widely observed in migraine (25–27), attention-deficit hyperactivity disorder (ADHD) (28, 29) and depression (30, 31) drug trials but to date, there are few experimental studies (32, 33) investigating the mechanisms underlying the PE, especially in very young children. Conceivably the PE plays a critical and unappreciated role in child health. The PE in these cases is associated with similar underlying mechanisms—just not always intrinsic to the patient.

Little attention has been placed on the effects a placebo treatment exerts on the people surrounding the individual being treated, i.e., the different entities (or proxies) inherent to the treatment environment that may have a direct or even reciprocal communication channel with the patient, such as clinicians (see Figure 1, Arrow A), family members (Arrow B) and caregivers (Arrows D) surrounding the treatment setting or online medical advice (e.g., Arrow E). In this narrative review we will examine what is known about “placebo by proxy” (PbP), and its inverse, “nocebo by proxy” (NbP), in treatment outcomes in children, as well as its clinical implications.

The concept of the PbP was first introduced in 2011 by Grelotti and Kaptchuk, who describe PbP as the positive effects a placebo treatment exerts on the people surrounding the individual being treated, e.g., family members, caregivers and clinicians (1). Proxies often feel better due to the mere fact that an individual is receiving medical care, a response regarded by the French anthropologist Claude Lévi-Strauss as the global “sense of security” which is critical for the social group's existence (34). PbP is also being described as having the potential to influence evaluation of treatment outcome, especially if proxies are exposed to encouraging objective signs displayed by the patient (35). Grelotti and Kaptchuk continue with the idea that perceptions or misperceptions among the parents may act as a contributing factor to the placebo response seen in children with treatment resistant epilepsy (36).

The first example for a PbP without being classified as such can be seen in patients with Alzheimer's disease (37, 38), where caregivers often report clinical symptoms on behalf of the patient and thus may influence treatment outcomes. For instance, it has been found in patients with Alzheimer's disease that negative caregiver bias (compared to self-report) at baseline, predicts and may even be considered a risk factor for developing apathy within a year (39). This example may illustrate biased reporting by the proxy that has a bearing on the study outcomes, originating in the caregiver's (negative) feeling and thoughts, which may subsequently impact the patient and study findings.

The role of the observer (i.e., proxy) in treatment outcome and the interpersonal alliance has also been studied in the context of pain, showing the importance of the proxy in motivating the patient to taking steps of self-caring behavior in their own healing process (40). This bond which underlies communication about pain projections is dynamic and subject to change over time due to learning processes by both the patient and the proxy. Besides expectation, two more modifying factors are believed to drive proxy's behavior ultimately altering patient's experience and the long-term coping with chronic pain: stigma and validation. The former is the suspicion raised by the observer to the invisible pain and its debilitating effects (41), and the latter, the inverse of stigma, is when the observer gives legitimacy to one's pain (40). In this narrative review, we take this definition of PbP one step further, building on Grelotti and Kaptchuk's definition, to include the impact of PbP (and NbP) as it transforms to affect the treatment outcome for the patient or child being treated (Figure 1, Arrow B), not just the people surrounding the individual being treated. We define PbP as the positive effects a placebo treatment exerts on the people surrounding the individual being treated, e.g., family members, caregivers and clinicians or the positive effect these proxies convey to the individual being treated resulting in a positive clinical outcome.

In contrast to a placebo response, a nocebo response is considered the worsening of a symptom after the administration of an inactive intervention, highlighted by increased pain observed in the context of placebo analgesia studies (42). Thus, the course of developing an adverse effect e.g., apathy following a caregiver's bias report as described above (39), can be considered an NbP. Correspondingly, we define NbP as the negative effects a nocebo treatment exerts on the people surrounding the individual being treated, e.g., family members, caregivers and clinicians or the negative effect these proxies convey to the individual being treated resulting in a negative clinical outcome. Other negative effects could occur in this regard but have yet to be systematically investigated; for example, when an ineffective treatment is continued only because proxies feel better about it or sense commitment to a certain treatment or clinician (see Figure 1, Arrow C). It also makes sense that a proxy will feel worse following an individual's treatment, which elicits side effects when the proxy perceives it as ineffective. A proxy may as well experience negative feelings stemming from the loss of secondary benefits such as extra attention, gratitude of the patient, or necessity. How this affects the patient's treatment directly or in the long run has yet to be examined, at least in placebo research.

This paper will review the PbP concept and its broadness and extended arms (depicted in Figure 1), provide evidence for its presence in children, introduce the concept of NbP, and offer suggestions for future research that further our understanding of the role of the proxy in both promoting or distracting from treatment benefit in children. Increasing an appreciation of the PbP and NbP phenomena and the role of the proxy in child healthcare should improve research study design and ultimately harness them to improve clinical child healthcare.

### Learning Processes in the Treatment Environment

The PE can arise from either conscious or unconscious mechanisms (4, 43–47) and there is comparable evidence of these processes in children (16, 24). Regardless of the chemical effect of a medication itself, placebos have been shown to mimic the activity of pharmaceutical agents given for the treatment of a wide range of conditions such as pain (48), depression (49) and Parkinson's Disease (PD) (50). Conscious cognitive and emotional factors such as anticipation (51), meaning (52, 53), faith (54), trust, belief (55), and hope (56), were shown to greatly differ between individuals and alter clinical outcomes. In contrast, unconscious factors such as the active component of the pill result in unconscious physiological changes, i.e., a conditioned response. This presumably reflects Pavlovian (or: classical) conditioning whereby, after repeated pairings between a conditioned stimulus (the color and shape of the pill) with an unconditioned stimulus (active component), the conditioned stimulus alone can generate a clinical effect. For instance, individuals who experience headache, and who regularly consume aspirin, are likely to link the pill's color, shape and taste to the relief felt afterwards. Following repeated pairings, a white, round and bitter pill resembling a known analgesic such as aspirin, could also elicit relief.

Thus, the placebo response may arise from learning whereby numerous nonspecific contextual factors such as white coats, syringes and nurses can come to function as conditioned stimuli as well (57). Moreover, neutral stimuli associated with relief in symptoms, e.g., the caregiver, the physical examination or the prescription of medicine may even procure positive and desirable healing properties. Positive vs. negative previous clinical experience has been shown to affect the magnitude of placebo analgesia. Following placebo administration, Colloca and Benedetti (58) reduced pain stimulus in a hidden manner to make patients “sense the effectiveness” of an analgesic treatment. This procedure elicited stronger and more lasting placebo analgesia responses compared to subjects who were not exposed to the manipulation (58).

Placebos have been reported to be more effective following an active treatment sequence compared to when given for the first time (59, 60), or when given to patients with severe dementia e.g., Alzheimer's disease (61–63); suggesting the role of memory in placebo-related learning. It is important though, to distinguish severe memory deficits from severe cognitive deficits, as the latter do not abolish expectation of clinical benefit. A recent study in intellectually disabled patients has shown their susceptibility to the certainty of receiving genuine medicine (64). The assessment of both active and inactive treatment effects is challenging not only in children, but also in adults with cognitive disabilities. A meta-analysis of 22 studies in adults with genetically determined intellectual disability (e.g., Prader-Willi or Down syndrome) tested placebo response rates when determined by either proxy or objective measures. Higher placebo responses were demonstrated in individuals with higher IQ, as well as in younger patients (65). Hence, conscious expectation is necessary for placebos to “work,” playing a major role, even in the presence of a conditioned stimulus.

To date, only a few studies can be termed “clinical trials” in placebo research, where participants were intentionally treated with placebos as treatment and appropriate control groups were included to control for other effects. More recently clinical trials designed to investigate the PE using an open-label placebo design (66) have been used to investigate a placebo treatment response within a psychosocial context including the participant's experience, expectation and feelings (67).

Furthermore, experimental studies found that placebo effects can be elicited through explicit social observational learning in laboratory conditions. A case in point, a person who observes a putative effective treatment in another person shows a similar placebo effect when treated with the apparently similar placebo treatment (68, 69). Implicit social learning of placebo effects could occur through observation of treatments of other persons in everyday life; for example, when children observe that white little pills decrease headache when their mothers took aspirin, and afterwards white little placebo pills decrease headache in children, too. However, this form of implicit learning is difficult to investigate, but could be estimated when placebo effects are induced and compared in family members. A recent experimental study induced conditioned placebo analgesia in both mono- and dizygotic healthy twins who grew up together and found no correlation of placebo effects within twin pairs, but a significant correlation of the PE with the conditioning effect. Conceivably, individual learning seems to play a more important role than implicit social learning, at least when tested in healthy adults in the laboratory (70).

## Placebo by Proxy in the Literature

### Search Method Overview

**Figure 2:**
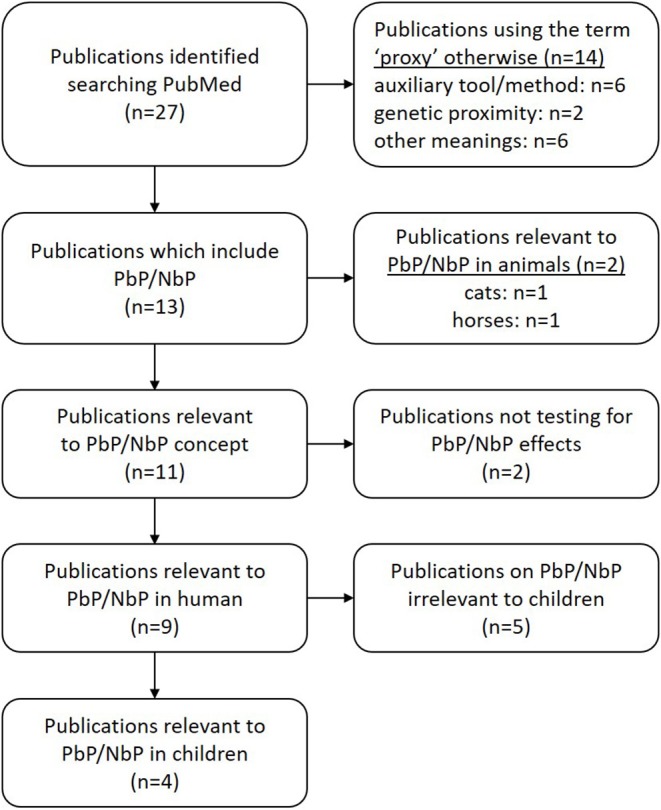
Paper identification and selection. A systematic literature search on PubMed using: placebo by proxy[Title] *OR* (“placebo response” *OR* “placebo effect” *OR* “nocebo response” *OR* “nocebo effect”) *AND* proxy.

The concept of PbP has recently received attention from a methodological point of view however little has been published on the PbP concept over the last 70 years (71). We have taken the following steps to ensure a qualitative review of the current knowledge on PbP. First, a comprehensive search of peer-reviewed articles (including data papers, meta-analyses, systematic reviews, reviews, commentaries, and several letters) was done using the Journal of Interdisciplinary Placebo Studies (JIPS) literature database (https://jips.online) to inform us about relevant keywords in this field. This database comprises ~4,500 articles (on January 2020) pertaining to the placebo effect/response which were hand-selected by PE and KW from PubMed on a weekly basis (71). Literature search is done based on the keywords “placebo” and “nocebo,” and relevant articles dealing with the placebo effect/response and the nocebo effect/response are selected and included in the database. This informative search revealed that the term “proxy” only seems to be a valid search term for a full literature search. In a second step, a systematic literature search was performed via the PubMed database (US National Library of Medicine, Bethesda, Maryland), crosscutting placebo by proxy[Title] *OR* “placebo response” *OR* “placebo effect” *OR* “nocebo response” *OR* “nocebo effect” with (boolean *AND*) the term “proxy,” resulted in 27 studies of which 14 used the dictionary meaning of the word “proxy,” often to depict an auxiliary tool or method or genetic proximity. This resulted in the identification of thirteen papers which included PbP/NbP—two in animals (cats, horses). Two papers used the term “proxy” acknowledging the existence of an auxiliary person in the treatment environment, but were not testing their effect. Only nine papers studied the PbP/NbP concept in human, five reported outcomes in adults, and four in children (72–75) (Figure 2). We further scanned the reference section of each article in order to look for additional publications but found none. Studies pertaining to PbP in adults will not be discussed further in the scope of this review.

### Placebo by Proxy in Children—Indirectly Measured

A placebo effect cannot be discussed without considering participants' understanding on the efficacy of medical treatment. For example, an individual's attitude to treatment has been shown to reflect previous medical experience (76, 77). Conscious cognitive elements e.g., in medical treatment (55), proper knowledge on the condition (55, 78) and actively affecting treatment decisions (79), all play an important part in engagement with treatment and clinical responses.

Descriptions of symptoms, particularly in self-appraisal conditions such as anxiety or pain, are associated with subjective and ambiguous self-report in children, often challenging an objective evaluation of treatment responses. Evaluating treatment responses in children requires from proxies (parents, relatives, caregivers) to make critical and timely judgements and interpretation of behaviors; when most of our knowledge comes from placebo-controlled randomized trials testing drugs rather than nonspecific effects. Whether symptom improvement is related to the expectation of a treatment rather than the active compound or medication is often difficult to determine in children where parents or other caregivers play critical roles in care and presumably in treatment responses. Young children, who have yet to develop sufficient language skills, solely depend on their parents. In fact, parents are expected to make decisions for their children without being fully informed about symptoms or being able to assess treatment outcomes due to their child's premature and limited communication repertoire. Thus, it is not surprising that parents and other proxies play a critical role in evaluating children's responses to medical treatment. Expectation of the parent (or other proxy) may contribute to the impact of a placebo or treatment itself, thereby contributing to a PbP. This can occur when a child's response to therapy is affected by the behavior of others who are aware of the therapy. In this sense, the placebo effect could operate indirectly by producing changes via how proxies themselves behave toward the child, which in turn leads to behavioral and symptomatic changes.

In their seminal paper, Grelotti and Kaptchuk base their observations of the PbP among individuals who rely on others to make treatment decisions because of inherent developmental, cognitive or communication limitations, such as the elderly with dementia or children (1). The authors argue that antibiotics which are often overprescribed for children only to meet parents' wishes and concerns (80), operate as impure placebos. Proxies' influence on placebo responsiveness may also be responsible for differences in expectancy reports seen between doctor and patient-reported outcomes, especially in depression (81). The notion of the PbP has been examined, albeit indirectly, in a variety of child health settings where parental expectancies appear to have a significant influence on reports of child behavior, parent–child interactions, and treatment responses, such as shifts in expectancy and frequency of health related-visits (30, 31).

In a classic study testing the effect of parental expectations on reported negative effects of sugar on children, mothers were told that their children have been given large sugar doses (experimental group) or placebo (control) when they were all actually given placebo (aspartame). Mothers in the experimental group, who were told that their sons received sugar did report their sons to be more hyperactive compared to the control mothers, suggesting that parents would rather attribute their children's high activity levels to an external and controllable factor such as “sugar” rather than to internal and complex origins e.g., psychological or behavioral problems (82). It could also be the case that mothers who “knew” that their child has received sugar affiliated their child's behavior with hyperactivity. In addition, mothers in the sugar expectancy group used more control and restraint toward their sons, who in turn showed lower activity (indicated by a wrist actometer) than their peers in the control group. This demonstrates that reporting of behavioral sugar effects on children maybe in part the result of parents' perceptual biases. These mothers also demonstrated their sugar expectancies in their actions, i.e., maintaining higher proximity to their sons and commenting more frequently on their behavior to take control over them (82). This change can also be seen as a “self-fulfilling prophecy,” which is also well-established in teacher-pupil interactions (83).

### Placebo by Proxy in Children—Studied as a Placebo Effect

The effect of PbP *per se* has been studied in children and their parents for temper tantrums, acupuncture for postoperative morbidities, as well as in very preterm infants. Whalley and Hyland were among the first to investigate whether a homeopathic remedy (Bach flower), presumed to be a placebo treatment for temper tantrums would be affected by parents' beliefs and emotions (72). Even after accounting for interactions between the physicians and either the child or the parent over the phone, parental mood was associated with both frequency of their child's tantrums and severity of parental mood. Importantly, this might be the first test of the impact of PbP as most children in this study were not informed of the reason they were given the flower essence, and those who were, did not exhibit different behavior. The authors note however that parents may have altered their behavior toward their children due to their awareness of the treatment, and therefore may have contributed to the change in tantrums. While a child's response to treatment for tantrums could be associated with parental beliefs, expectations and mood, it remains unclear whether a reduction in tantrums was due to objective changes in child behavior, changes in parental perception, or both. The relationship between parents' daily mood and child tantrums should be considered, as it remains unclear whether this effect was mediated by altered parents' behaviors toward the child. These findings highlight the importance of the perceived meaning of a treatment response, which may underlie the source of the placebo effect (53).

In a study of parental expectancies before acupuncture treatment for postoperative symptoms in children, compared to post-acupuncture expectancies, Liodden et al. (73, 75) did not find an association between the children's symptoms and parents' expectations. However, they did report that positive changes in parental expectation were reflected in better (less) post-operative symptoms in the children (75). In this study, anxious parents tended to change their expectancy in a positive direction while treatment was ongoing, which may have led to reduced postoperative vomiting in children. The investigators suggest that parental anxiety could be assessed preoperatively and perhaps managed to elicit PbP effects. While parental anxiety has at time been observed as a barrier, it could be considered as a possible facilitator of improved child outcomes in an acute care setting (75).

A placebo-controlled study investigated the neuroprotective effect of early high-dose recombinant human erythropoietin in very preterm-born infants (74). Burkart et al. examined whether parent's belief that their infants had received the drug vs. placebo made a difference in long-term development (74). Children of parents assuming that their infant had received verum showed a small but significant difference in IQ at 5 years of age compared to placebo, however this difference was determined as clinically insignificant. School teachers have also been shown to be essential proxies when rating the impact of therapy for childhood behavioral disturbances such as ADHD. One study reported that both parents and teachers tend to have a positive bias when evaluating ADHD symptoms in a child who they believe has been given medication (84). The authors suggest that the change in the caregiver's perception of their child's behavior following the administration of medication, was very similar to an expectancy effect on the child receiving treatment.

## Nocebo by Proxy in Children—A Useful Concept

The impact of the proxy can also go the other way and the “proxy” effect can potentially exert a negative or adverse effect on a child's treatment outcome, whereby a proxy feels worse leading to a NbP effect. Extending the Grelotti and Kaptchuk definition of PbP, this would comprise the negative effects that a placebo treatment exerts on the child via the beliefs or perceptions of people surrounding the child being treated, e.g., family members, caregivers, and clinicians, not just the impact on the caregiver (1). Such an effect could occur when a proxy observes that a treatment is ineffective or is perceived as causing adverse effects leading the patient (i.e., child) to experience negative side effects. While the NbP phenomenon in children has yet to be systematically studied in placebo research, we can obtain some indication that it may be present from studies of a parent's response to a child's pain or temper tantrums, reflecting that a parent's behavior acts as a significantly influential factor on children's pain and function. There is substantial evidence suggesting that maladaptive parental responses to children's pain, such as reassurance, solicitous, and protective parenting behaviors, increased children's susceptibility to adverse outcomes in both clinical pain populations (85–87) as well as for experimentally induced pain (88, 89). A case in point, in a study of parental response to children's chronic pain examining the moderating impact of children's emotional distress on the perception of symptoms and disability, patients' parents assessed parental responses to their children's pain. Where parents responded to their child's pain with criticism, discounting of pain experience, increased focus to pain, or granting of special privileges, children appeared to have higher levels of emotional distress, increased disability and somatic symptoms. Among youth who infrequently use passive or active coping strategies, higher parental protective behavior was associated with higher levels of disability and somatic complaints (87). Similarly, parental solicitous behavior was associated with more child distress and greater disability (90, 91). Studies of acute pain have demonstrated that children require more restraints and express high levels of fear when parents provide reassurance, compared with distraction during immunizations (92–94). Interestingly, one study found a relation between parenting responses and parental distress, such that parents who were trained to reassure their children during an immunization procedure were more distressed after the procedure was completed (93). Importantly, studies of chronic pain have specifically linked parental protective responses to high levels of children's functional disability (95).

## Discussion

### Clinical Implications

As clinicians, parents and their children are active participants in treatment outcomes, there needs to be a sensitivity to the possibility that a treatment response may arise from processes that reflect PbP and/or NbP. At present, very limited attention has been paid to potential practice, training, and ethical implications of parent responses—be it placebo or nocebo—that contribute to a treatment response in children. It is conceivable that what clinicians communicate to parents about treatments might affect treatment outcomes via enhancement of parental expectancies, thereby (potentially) enhancing placebo and nocebo effects in children. Given that words and behaviors matter (42, 96), parents should be made aware that their own responses can influence their children's health outcomes and this raises critical questions of whose responsibility is it to educate parents about their critical roles, for better and worse. Thus, formulating a structured clinical approach that harnesses a parent's or clinician's expectations of treatment benefit (i.e., the placebo effect) via attending to symptoms (solicitous or protective), granting permission to avoid regular activities or saying “this medication may not work, but it's worth trying” vs. “this treatment has been shown to work with other children and I think it will help you.” Appreciating different directions of communication, the variety of ways that proxies can take part, and the direction and intensities that each player contributes to the patient's experience, should be considered throughout the therapeutic period (Figure 1). Our challenge is to identify these phenomena and harness them to improve research design, clinical practice and training for clinicians and parents alike.

### Future Research

Appreciation of treatment expectations and behavioral roles of the “proxies” represented by parent, caregiver, and peers and how they contribute to shaping treatment responses for better and worse should be essential components of clinical care and research design, but given the paucity of reports of PbP/NbP, this requires careful empiric observation of the components that comprise the clinical encounter. Then using this knowledge could be used to inform study designs that manipulate parental/caregiver expectations, beliefs or behavior about treatment effects to change treatment effects. Given that the dyadic nature of of PbP (and by extension the NbP) transforms the treatment outcome itself for the patient or child being treated, not just for the people surrounding the individual being treated, study design needs to consider the multidirectional nature of parental influences, i.e., path analysis. This could include assessment of parental beliefs, mindset attitudes, and expectations guiding their perceptions of the treatment received by their child. Further, it is conceivable that perceptions of competence and empathy also influence placebo effects in this setting (97, 98) whereby some parents are better able to enhance placebo effects via competence/empathy cues. The scarce data available brings the necessity in future research of placebo studies and parents' role in their children's treatment.

### Limitations

To date we know very little about how parental/caregiver expectations of treatment for a child affect that treatment outcomes for their child/patient. What we know is that parents/caregivers matter in treatment outcomes, but how this operates and how this can be utilized for clinical benefit remains to be determined. To understand the inherently dyadic roles parents play in the treatment outcome, as well as the vulnerability in this intricate relationship, parents' perception of the treatment outcome or of their child's pain may change unexpectedly over the course of treatment which may be at times maladaptive behavior/unintentionally not in favor of their child, such as overprotection resulting in increased child's pain.

Key ethical considerations are required with respect to what clinicians might communicate to parents about treatments to enhance parental expectancies, thereby (potentially) enhancing placebo and nocebo effects in children. Considering the PbP or NbP phenomenon in both clinical practice and research requires recognition of critical ethical considerations, however, there are no reasons to believe these would be different from the key considerations outlined by Blease (2). These include avoiding: (1) deceptive practice, (2) risk of conveying an impression that all the symptoms are “in your head,” and (3) the notion, that may be inherent to a placebo benefit, constraint of help-seeking behavior (i.e., lack of faith in mainstream medicine that may be beneficial and necessary). Finally, to advance our understanding of the role of the proxy in evaluating treatment response in children, attention needs to be drawn to the impact of the caregiving setting for young children and children with developmental disorders that limit communication and cognition.

### Summary

The placebo by proxy effect is a complex dynamic interactive phenomenon which attempts to explain an individual's response to treatment arising from an interaction between the individual and an effect on proxies such as parents, partners, physicians, or even the media. In this sense, the PbP needs to include the impact of PbP (and NbP) as it transforms to affect the treatment outcome for the patient or child being treated (Figure 1, Arrow B), not just the people surrounding the individual being treated. In this sense, we define “placebo by proxy” in children as a “placebo effect by proxy,” namely where a proxy's belief or expectation of benefit leads to therapeutic benefits to the child associated with administration of a placebo in the context of individuals being treated. Placebo by proxy is an inherently reciprocal phenomenon possibly reflecting that when a proxy feels better (i.e., they convey a response to the placebo administered to the patient/child), they in turn behave differently toward the patient who in turn experiences symptom improvement. Such symptom improvements could reflect direct placebo effects, but in this context, could also originate from contextual factors affecting the proxy without excluding the possibility that it has also arisen from a patient's response to the treatment itself. Thus, both the patient and the proxy experience positive effects. Alternatively, this could also occur when an ineffective treatment is continued only because proxies “feel better” or are committed even to an ineffective treatment.

While the causal direction of effect (parent to child or vice versa) remains to be determined, these findings reflect critical ways in which parents can shape the way their children cope with and manage for example, chronic pain. Together, the current findings indicate that maternal behavior can have a direct impact on a child's pain report, highlighting the reciprocal dyadic contextual nature of a child's pain experience and supporting the importance of social learning factors in influencing children's pain experiences. Contextual factors clearly affect response to medications and so it is not surprising that proxies (such as parents) play role in treatment responses, considering that PbP is the positive effect of a placebo and not dissimilar to the negative effect of NbP, that occurs within the social environment of a treated individual.

For children, such proxies might include family members, caregivers, healthcare providers and friends, as well might include online medical advice (Dr. Google), via parents. This notion implies that PbP and NbP can take the shape of a large variety of entities inside and outside the clinical setting, be it directly related to human interactions or indirectly via social media or the internet. Additionally, there can be complex reciprocal effects between placebo by proxy and placebo effects, i.e., patients who receive treatment are receptive to the behavior of their proxies, who tend to respond emotionally and straightaway receive medical attention. In an active voice, proxies are often involved in treatment decisions or even decide for the patient based on their observation and interpretations.

Presumably, when a proxy feels better, they may in turn behave differently toward the patient which may affect symptoms. Such symptom improvements are themselves due to placebo effects as they do not originate from the treatment but from contextual factors. The placebo response in children and adults is not necessarily determined by the same factors or perceived in the same ways. Thus, it is possible that the improvement (or potentiation of adverse outcomes) we witness in children may be mediated by a placebo (or nocebo) response experienced by the parent or other proxy, rather than that experienced by the children themselves.

## Author Contributions

EC, TO, KW, JK, and PE contributed to the conception of the manuscript and reviewed the literature. EC, TO, KW, and PE drafted the manuscript. JK and PE critically revised the manuscript.

### Conflict of Interest

The authors declare that the research was conducted in the absence of any commercial or financial relationships that could be construed as a potential conflict of interest.
